# Spiritual Self-Care Management for Nursing Professionals: A Holistic Approach

**DOI:** 10.1177/08980101211034341

**Published:** 2021-07-21

**Authors:** Håkan Nilsson

**Affiliations:** University West

**Keywords:** meditative, spiritual, self-care, body scanning, prayer, walking

## Abstract

Among all the groups and occupations that have been affected by the global pandemic, nursing professionals stand out as having been particularly hard-pressed due to the dramatic increase in the need for their services. Given the rising tide of coronavirus disease 2019 patients who require specialized medical treatment as well as the millions of others that are lining up for vaccinations, it can be assumed that many nursing professionals have had to endure such things as longer working hours, tighter schedules, and the intensity of a work environment in which failure of care and multiple deaths are the daily fare. This article proposes that nurses can avoid such severe consequences by taking up a regime of enhanced self-care management that enables them to achieve psychophysical balance and wellness. Three practices are highlighted in this regard: prayer, meditative walking, and the mindfulness practice of body scanning. Our suggestion is that these coping strategies will be both beneficial and healthful for nursing professionals in terms of enhancing their spiritual/existential resilience and meeting their own need for consolation as they navigate in an extremely difficult and demanding work environment.

Medical researchers throughout the world are currently placing great emphasis on mastering coronavirus disease 2019 (COVID-19). While several pharmaceutical companies have released vaccines in an attempt to curb the spread of the virus, it will take some time and the accumulation of solid statistical data before we know how effective those vaccines are in terms of permanently protecting inoculated individuals from infection and death. In the meantime, the number of deaths, which currently stands at around four million worldwide, continues to climb. In light of this reality, nursing professionals must be able to cope with a tremendous amount of stress, anxiety, and emotional/psychological pain. Nurses, for example, must regularly endure such things as longer working hours, tighter schedules, and the intensity of a work environment in which failure of care and multiple deaths are the daily fare. As a result, the risk of nurses suffering from burnout syndrome as well as physical and mental fatigue has been greatly heightened. This article proposes that nursing professionals can avoid such severe consequences by incorporating a regime of self-care management which enables them to achieve psychophysical balance and spiritual wellness. Indeed, decades of general research on the relationship between spirituality and health (or wellbeing) indicate a number of positive effects—e.g., lower rates of cancer, heart disease, alcoholism, and mental illness as well as improvements in the quality of life (Koenig, 2012; Koenig et al., 2001; Park, 2007; Rao et al., 2015; Thoresen & Harris, 2002). Given the large amount of data on this subject, there is good reason to suppose that nursing professionals will greatly benefit from setting aside time each day for self-care practices which encourage self-reflection, freshen the spirit, and enable the continued administration of respectful, loving care. In terms of the means by which nursing professionals can strengthen and revitalize their own physio-psycho-spiritual wellbeing, this article focuses on three helpful practices: mindful body scanning, prayer, and meditative walking. Before providing an elaborate description of these three practices and how they can be applied within the nursing profession, we will attempt to conceptualize and contextualize the terms “spirituality” and “self-care management” as they relate to nursing in particular and holistic health in general.

## The Term Spirituality

Merriam-Webster Online Dictionary defines the word “spirituality” as “sensitivity or attachment to religious values” as well as “the quality or state of being spiritual.” Spirituality, however, is a complex term that can take on a diversity of meanings in accordance with each person's individual experiences. Anandarajah and Hight (2001) note that spirituality contains cognitive, experiential, and behavioral aspects. The *cognitive aspect* is said to involve our search for meaning, purpose, and truth as well as the beliefs and values by which we live. The *experiential aspect* is said to involve feelings of hope, love, connection, inner peace, and comfort, and can reflect the quality of our inner resources, our ability to give and receive spiritual love, and the caliber of our relationship with ourselves, the community, the environment, the natural world, and the transcendent (God, cosmic consciousness, etc.). Finally, the *behavioral aspect* is said to involve the manner in which we display or act upon our spiritual beliefs and inner spiritual consciousness (Anandarajah & Hight, 2001).

While keeping these various definitions in mind, for purposes of this article we have adopted an understanding provided by Buck, which allows for either a sacred or a secular/existential approach to spirituality. Buck (2006) notes that “most human experiences seek to transcend self and find meaning and purpose through connection with others, nature and/or a Supreme Being, which may or may not involve religious structures or traditions” (p. 289–290). Following along these lines, the three self-care practices outlined in this article—that is, mindful body scanning, prayer, and meditative walking—can be approached in either a scared manner (involving the search for meaning and purposes that are oriented around God and a given religious tradition) or in a secular/existential manner (involving the search for meaning and purposes that are *not* oriented around God and a given religious tradition). Although it is difficult to entirely remove the notion of God from the notions of either religion or spirituality (Domingo-Osle & Domingo, 2020), the more nuanced distinctions between these two orientations will become clear as we proceed, as will the strong connection between spirituality and holistic health—something that can potentially contribute to personal and relational wellbeing and bring solace, hope, and existential resilience^1^ to persons in need. Having thus established a working understanding of the term spirituality, we will next attempt to conceptualize and contextualize “self-care management” within the framework of the healthcare setting.

## Self-Care Management: Concept and Context

White (2013) has noted that self-care practices have not been “socialized into nurses’ way of thinking or into their work environment.” Along the same lines, Auser et al. (2021) argue that spiritual self-care practice remain underdeveloped and unexamined within the nursing profession. Instead, modern nursing is primarily defined by an outward commitment to patient care—an ideal established by Florence Nightingale (1820–1910), who played a major role in the founding of the profession. While attending to the needs of patients is certainly at the core of what it means to be a nurse, as the pressures and demands on the profession continue to mount, attending to their own self-care through practices like mindful body scanning, prayer, and meditative walking becomes equally important. In the literature, the term self-care has been defined as “engaging in health-related activities and using health-promoting behaviors to adopt a healthier lifestyle and enhance wellness” (Dossey & Keegan, 2013, p. 828). In terms of the nursing profession, it would be logical to assume that unless nurses regularly see to their own physical, psychological, and spiritual/existential wellbeing, their ability to provide the highest level of patient care will gradually diminish—a conclusion also acknowledged by Nightingale herself (cf. Riegel et al., 2021). The phrase “health-related activities” in the above definition can be interpreted in the broadest sense to include the three dimensions of health just mentioned along with the fourth, social, dimension. In this article, when speaking of self-care, we include all these vital dimensions of health, but primarily focus on the three activities mentioned above, namely mindful body scanning, prayer, and meditative walking, all of which contribute holistically to a healthier lifestyle and enhanced wellness. The process of including such practices as part of a nurse's job description, however, faces at least two challenges: (a) the ever-increasing time and patient-care demands of the profession make it challenging for nurses to take time for what some describe as unrelated or extracurricular activities; (b) there seems to be a bias within the profession against the use of contemplative practices to enhance health and wellbeing (cf. Narayanasamy & Narayanasamy, 2008). This notwithstanding, a rapidly growing body of evidence indicates that practices like mindful body scanning, prayer, and meditative walking constitute beneficial forms of nonpharmacological intervention which can contribute to physical, psychological, social, and existential self-care (Irving et al., 2009; Lubinska-Welch et al., 2015; Narayanasamy & Narayanasamy, 2008; Puchalski & Guenther, 2012; White, 2013). Although Asian holistic healthcare practices such as acupuncture, Ayurveda, and Tai Chi have gained much Western acceptance and attracted further research, Dean (2001) points out that most Western professionals still view Asian healthcare management and Western healthcare management as two distinct disciplines, with hardly anyone proposing an integrated Asian-Western approach to healthcare.

Providing nursing professionals with the opportunity to make mindful body-scanning, prayer, and meditative walking primary self-care tools brings holistic health into the healthcare setting, increasing nurses’ ability to cope with one of the most demanding jobs on the planet. This particular approach to self-care management also has been shown to enhance human qualities such as empathy and compassion, both of which contribute to the effectiveness of patient care. The following section discusses mindfulness in general as a self-care tool that can contribute to a more balanced work life among nursing professionals.

## Mindfulness

Mindfulness as a form of holistic intervention and therapy has shown considerable promise in the area of health care (Nilsson, 2016). Here mindfulness can be understood as a neutral self-regulatory tool that helps improve a variety of mental and physical conditions without the necessity of embracing a Buddhist perspective. It is an “evidence-based” practice that promotes holistic health and contributes to self-understanding (White, 2013). In the literature, mindfulness has been defined in various ways; here, however, we define it as *a particular type of social practice that leads the practitioner to an ethically-minded awareness, intentionally situated in the here and now* (Nilsson & Kazemi, 2016). The standard mindfulness regime consists of three practices: meditation, yoga, and body scanning. In this section, we discuss only body scanning, largely because it bears a strong resemblance to paradoxical relaxation training (Wíse & Anderson, 2010) and progressive muscle relaxation training, both of which are common relaxation methods among Western athletes (Jacobsen, 1924). This is not to say that nursing professionals should avoid introducing their patients to meditation and/or yoga as additional mindfulness regimes. In this regard it is important to be aware of the historical parallels between meditative spirituality (e.g., the works of Christian theologians such as Gregory and Augustine) and mindfulness (Hathaway & Tan, 2009; Sun, 2014). As Hathaway and Tan (2009, p. 169) note, “[t]he challenge is to find clinically useful ways of harvesting such sophisticated theological and philosophical reflections.”

Mindful body scanning involves setting aside time to go to the mental “gym,” and this entails dedicating a certain period each day to becoming more resilient and present in the body (Kabat-Zinn, 2013). Body scanning can be effectively practiced even for a short period of time, even while lying in bed at night or in the morning, and even while sitting or standing during breaks. There are numerous creative ways of bringing body scanning (or similar forms of spiritual relaxation and renewal) into one's life (Kabat-Zinn, 2005). Mindfulness practices, however, are best learned with the help of a trained mindfulness instructor. If they are educated in mindfulness, nursing professionals also can train interested patients to body scan; if not, they can direct them to a mindfulness center, where they can receive proper instruction. Body scanning contributes to self-care management in a way that enables practitioners to better handle their own physical tensions and disturbing thoughts/feelings. It also improves spiritual/existential resilience and brings comfort both to oneself and to those around one; here the application of these types of benefits to the nursing profession should be fairly obvious (cf. Lubinska-Welch et al., 2016; White, 2013).

The sort of concentrated relaxation and revitalization that mindful body scanning provides can be an important self-care tool for nursing professionals, especially as a means of coping with the demands of their profession and handling the highly stressful circumstances of their daily lives. Taking the time to develop a deep awareness of one's own body as well as learning to control one's breath can have a positive impact on feelings that are connected to stress and pain. Kabat-Zinn's (2013) expert assessment of mindfulness and mindfulness-based stress reduction (MBSR) is based on years of personal experience working with stress/pain patient groups. Regarding mindful body scanning, he writes:

Each time you scan the body, you are letting what will flow in flow out. You are not trying to force ‘letting go’ or purification to happen, which of course is impossible anyway. Letting go is really an act of acceptance of your situation. It is not a surrender to your fears about it. It is a seeing of yourself as larger than your problems and your pain, larger than your cancer, larger than your heart disease, and larger than your body and identifying with the totality of your being rather than your body or your heart or your back or your fears (p. 88).

Letting go, as Kabat-Zinn here emphasizes, is something that nursing professionals can bear in mind as they confront the daily challenges—the stress, the frustrations, the emotional highs and lows—entailed in treating COVID-19 patients. The following section examines the potential contribution of prayer to self-care management.

## Prayer

Throughout human history individuals have confronted various forms of suffering, and thus have sought consolation in a diversity of ways. For those that are religiously (or spiritually) inclined, prayer has served as a primary means of experiencing consolation, healing, and hope (cf. Narayanasamy & Narayanasamy, 2008). Prayer, moreover, has been acknowledged in both ancient and modern times for its contribution to the alleviation of illness and the promotion of good health (Chandramohan & Bhagwan, 2019). Sister André, a 116-year old French nun, serves a case in point; considered the second oldest known woman in the world, the sister contracted COVID-19, afterwards claiming that it was prayer more than anything else which enabled her recovery (Peltier, 2021). Along the same lines, Andrew Newberg et al. (2015) showed that intense Islamic prayer decreased frontal and parietal lobe activity, increased will power, and deepened feelings of connectedness to God. Although for most people prayer is conceived as invoking the intervention of a supreme being, it also can be understood more broadly as act which helps one connect to some sort of metaphysical reality or cosmic consciousness. In any case, prayer has undoubtedly played a crucial role in the history of the spiritual. This section examines prayer as either a spiritual-, religious-, or meaning-centered practice that contributes to self-care management and affords the practitioner the possibility of consolation, hope, and existential resilience.

For confirmation of the ubiquity of prayer in the modern context one need only turn to the music industry, which for decades has produced songs involving prayer—songs that have been heard by generations of listeners, which have soothed the soul and provided genuine relief during times of personal and societal crisis. Indeed, a quick Google-search reveals such titles as: Nat King Cole's *Sweet Hour of Prayer* (1959), Otis Redding's *My Lover's Prayer* (1966), Dionne Warwick's *I Say a Little Prayer* (1967), Jon Bon Jovís *Livińon Prayer* (1986), and Bruce Springsteen's *The Power of Prayer* (2020). These and other such songs indicate that music has the power not only to console, but also to change the way we perceive the world with the help of prayer. While these are examples of American artists, songs about prayer, and prayers which have been converted to songs, can be found in all cultures and times.

Prayer, in general, is most ardently employed during the most difficult and challenging times of our lives—times in which we experience death and loss (Walsh, 2015). And, of course, nursing professionals must confront the reality of death and loss on a regular basis, especially when working in the midst of the current pandemic.

Among the benefits derived from therapeutic prayer for nursing professionals is the ability to achieve emotional catharsis and/or spiritually cope with the pressures of tragic or painful situations (cf. Sohail, 2018). Sohail (2018, p. 936), for example, provides the following statement made by a Muslim woman: “Being a Muslim I have no doubt… that prayers are a way to talk to God (Allah). I have experienced that Allah helps me to fight with pain when pain killers stop working.” It can also be said that prayer, as a spiritual coping mechanism, helps to attenuate stress coming from healthcare settings like hospitals or clinics (Vasconcelos, 2010).

For Christian-oriented nursing professionals, there are prayers for all types of life circumstances, as one can learn by visiting websites such as Logan (2020) or Freeland (2019); and for those that are more secular in their outlook, there are neutral prayers, the content of which has no particular religious reference (Nursebuff.com, 2018; Vera, 2021). The following poetic statement provides an example of a prayer that is not specific to a given religion, but nonetheless emphasizes the virtues of wisdom and compassion, qualities which are valued by Buddhists, Christians, and even atheists alike (Vera, 2021):

Give me strength and wisdom,

When others need my touch;

A soothing word to speak to them,

Their hearts yearn for so much.

Give me the joy and laughter,

To lift a weary soul;

Pour in me compassion,

To make the broken whole.

Give me the gentle, healing hands

For those left in my care;

A blessing to those who need me.

This is a Nurse's prayer.

With regard to Buddhism, there is a type of Buddhist prayer known as “loving-kindness meditation” that can be employed by nurses, patients, and significant others who are inclined toward the Buddhist tradition. The practitioner begins to meditate while lying down or sitting in a particular posture (e.g., lotus, half-lotus or kneeling). After several minutes of silent meditation, the practitioner wholeheartedly contemplates the following four phrases: “may I be safe, protected and free from inner and outer harm; may I be happy and contented; may I be healthy and whole to whatever degree possible; and may I experience ease of wellbeing.” Each of these phrases is intended to be directed first toward oneself, then toward an important benefactor, then toward an intimate friend, then toward a neutral person, and finally toward an enemy. At the end, the meditator is supposed to direct all four phrases to all living beings (Nilsson, 2014).

Although prayer remains a controversial subject matter in the fields of social science and medicine, studies have shown that 25%–72% of respondents (e.g., clinical social workers) have prayed *for* clients, while 15%–43% have prayed *with* clients (Sheridan, 2010). Moreover, in a study regarding the self-care practices of 45 nurses, 53% reported praying on daily (or almost daily) basis (Lubinska-Welch et al., 2016). Austrian logotherapist Victor Frankl (1905–1997) also has acknowledge the importance of prayer for human existence. According to Frankl (2016), prayer is the highest form of the Buberian I-Thou relationship; and Swedish Methodist pastor Göte Bergsten (1945) once stated that in prayer the soul seeks and meets a Thou. Here an important distinction must be made between mechanical, routinized praying, in which one simply recites a prayer on automatic pilot without coming in touch with one's inner feelings or genuinely connecting with one's chosen object of prayer. This can be referred to as a “mindless” type of praying as opposed to praying which is truly, deeply, and profoundly “mindful.”

To be mindful when praying is to be intentionally present and attentive to the deity in the here-and-now—a relationship that is exemplified by the following passage from John (17:21): “That all of them may be one, Father, just as you are in me and I am in you. May they also be in us so that the world may believe that you have sent me.” This type of prayer can be considered truly transactional, meaning that one enters into a personal dialogue in which one pours his/her heart out to God (Narayanasamy & Narayanasamy, 2008, p. 243).

Through prayer, God (cosmic consciousness, etc.) becomes present as a comforting partner (Frankl, 2006). As the German theologian Friedrich Heiler (1932) nicely puts it: “Prayer is a direct manifestation of the perception of having a relationship with an immediate, personal God.” While prayer can be a powerful healing agent for both nursing professionals and their patients, it is ethically important for nurses to seek their patient's permission before attempting to introduce praying as a means of consolation (cf. Sheridan, 2010).

## Meditative Walking

Walking—that is, placing one foot in front of the other—is one of the oldest and perhaps most underrated activities in human history. Ever since humankind abandoned the trees for the ground humans have been using their legs to transport themselves for a variety of purposes. Back then, walking on the savanna was a means by which to survive from one day to the next, *finding* rather than *becoming* food. Ancient religions such as Judaism walked to the founding-point of their traditions. Called by God, both Abraham and Moses led the Jewish people in epic walks to the “Promised Land”; and both Jesus and Mohammed traveled constantly by foot to spread their teachings, as did the Buddha and the Hindu sage. Nowadays, of course, walking has been supplanted by various other means of transportation, thus becoming more of a lifestyle—a way to live a healthy life. Today, anyone with a smartphone knows that “10,000 steps a day” is the count that leads to fitness in the 21st century.

Walking, however, has served purposes other than mere survival or health. Historically, a plethora of famous authors and philosophers have used walking as a means of gaining inspiration and refreshing their creativity. The existentialist and urban “*flaneur*” Sören Kierkegaard (1813–1855) never missed a day's stroll round the beautiful city of Copenhagen, during which he would take in the sights and sounds of the surrounding milieu and incorporate them into his novels. In a letter to his niece Henriette Lund, Kierkegaard writes: “Above all, do not lose your desire to walk: every day I myself walk into a state of wellbeing and walk away from every illness. I have walked myself into my best thoughts, and I know of no thought so burdensome that one cannot walk away from it” (Kierkegaard quoted in Minshull, 2019, p. 3). The French philosopher Jean-Jacques Rousseau (1712–1778), who shared Kierkegaard's passion for walking, would wander through the streets of Paris, reflecting on the human condition. Rousseau wrote: “I like to walk at my ease, and stop when I like. A wandering life is what I want…” (Rousseau quoted in Gros, 2015, p. 80). In his autobiography *Les Reveries of the Solitary Walker* (1776), Rousseau tells how he reflected on his life and personal shortcomings while walking about Paris with a mindset resembling the sort of stream-of-consciousness thinking that authors like James Joyce (1882–1941) and Virginia Woolf (1882–1941) would employ in their writings some 100 years later.

Across the Atlantic, the American poet William Wordsworth (1770–1850) is known to have composed most of his works while walking and rhythmically murmuring poetic lines (cf. Gros, 2015). Indeed, one of his most famous poems, “Lines Written a Few Miles Above Tintern Abbey,” was composed during a pedestrian journey through Wales in 1798 (cf. Sohail, 2018). Then there is fellow countryman Henry David Thoreau, who Coverley (2012) called the greatest of American walkers. In his essay, appropriately titled “Walking” (1862), Thoreau describes a naturalistic landscape far removed from urban life. For Thoreau walking was a means of escaping the crowded hustle and bustle of the city to find solitude and freedom: “…if you are ready to leave father and mother, and brother and sister, and wife and child and friends, and never see them again—if you have paid your debts, and made your will, and settled all your affairs, and are a free man, then you are ready for a walk” (Coverley, 2012, p. 116).

Apart from all the purposes outlined above—that is, survival, health, inspiration, creativity, solitude, and freedom—walking also can serve nursing professionals by enabling them to release job-related tensions and pressures as well as to refresh and restore their energies both prior to and after work—or even during daily breaks. For such purposes meditative walking can help nurses to unwind, relax in the present moment, and strengthen their existential resilience (Lubinska-Welch et al., 2016). In keeping with this view, Sheehan writes: “But more than anything, that hour… [of walking] is for ideas and principles, for meditation and contemplation. Somehow in the relaxation, the letting go, we arrive at a state that Heraclitus described as ‘listening to the essence of things’. We open up to the world” (Cited in Koski, 2015, p. 104).

But what of nursing professionals who are so pressed by the demands of their daily workload that they simply have neither the time nor the energy nor the inclination to walk, whether before, during, or after work? Here we can assume that managing their regular rounds while simultaneously handling the steady influx of COVID-19 patients has made it extremely difficult for nurses to employ meditative walking as a means of recovery and renewal. For such nurses the silver lining is that they already do a great deal of walking in the ordinary course of their day, as the study “How Far do Nurses Walk” has shown (Welton et al., 2006). From this study we discover that American nurses walk an average of 4–5 miles during a regular 12-hr shift; this can be compared to Americans in general, who have been found to walk an average of 2.5–3 miles during a regular 18-hr day. Nursing professionals themselves can look upon this statistic as either an unpleasant consequence of their profession or an opportunity to squeeze in moments of meditative walking while, for example, moving from point A to point B along their ordinary route and being mindful of things like rapidity of pace, proper breathing—and even the addition of prayer (cf. Wall & Nelson, 2003). During my own meditative walks I like to inwardly say “Lord Jesus Christ, Son of God” while inhaling through the nostrils and “Have mercy on me, a poor sinner” while exhaling through the mouth. This type of “heart breathing” deeply moves the breath from the throat to the stomach through the vagus nerve, which controls the parasympathetic nervous system. The heart slows down, the body calms down, and I somehow become better able to recognize, and even cope with, adversity (Nilsson, 2016). By walking while repeating this “Jesus Prayer” in the manner described above, one can achieve an intense state of concentration (cf. Gros, 2015).

Among other things, meditative walking can help to revitalize nursing professionals by releasing bodily tensions, clearing the mind, grounding them in the present moment, and restoring their sense of inner peace. As an aid to meditative walking one also can use “inspirational spiritual cards” (for the religious) or “inspirational secular cards” (for the nonreligious). In my own experience as an enthusiastic walker, such cards are best used when walking (or wandering) longer distances (7–12 miles), preferably through the woods or some serene location which allows for forest bathing (*Shinrin-yoku*). One can walk in total solitude or silently with a friend. Forest bathing, by the way, is known to be an effective means of managing stress because it increases parasympathic and decreases sympathetic nerve activity (cf. Park et al., 2010). Regarding the cards, they are meant to stimulate mindful reflection during walks. By combining the physical journey of walking with the inner spiritual journey of meditating on the cards, one can relieve stress, quiet disturbing thoughts, and restore emotional balance. Because I am a Christian, my personal cards (seen below) draw inspiration from the Bible. Obviously, the card's content will change according to one's chosen faith, whether religious or secular, and may include verses from the Quran, the Bhagavad Gita, the Suttas, Mahatma Gandhi, Walt Whitman, William Shakespeare, or even one's wise old grandmother.

## Examples of Both Sacred and Secular Cards for Meditative Walking

### Bible Card 1 (2. Sam 22:37)

*You provide a broad path for my feet, so my ankles do not give way*.

Before setting off, read the card slowly; reflect on the verse's meaning and how it relates to your life circumstance. What can you learn from this verse? If you feel inspired by reading the verse, ask yourself why? What do you require so as to feel solid ground under your feet? Is there a particular prayer you wish to send to God? Keep the verse in mind, contemplating it repeatedly during your pedestrian journey. Once you have reached your destination, sit down and see what comes to mind.

### Secular Card 1

*How have my family members, friends and colleagues opened the way for me?*

Before setting off, read the card slowly; reflect on the sentence's meaning and its relevance to your life circumstance. Open your heart and let family members or significant others enter, filling your heart with joy and gratitude. If you face problems or issues with a particular family member or significant other, how can this sentence be used to overcome them? Keep the sentence in mind as you walk and ask, “what can I do that I have not yet done to improve the situation?” Once you have reached your destination, sit down and see what comes to mind.

## Conclusion

In closing, it can be said that a self-care regime which encompasses mindful body scanning, prayer, and meditative walking can help nursing professionals to cope with difficult job-related circumstances by mitigating stress, anxiety, and daily institutional adversities. This article advocates for a holistic, multidimensional approach to medicine that addresses, in totality, the physical, mental, social, and spiritual/existential wellbeing of the individual. In this regard, mindful body scanning can be seen as a technique which addresses both the physical and the mental dimension by strengthening the body and revitalizing the brain. Prayer, of course, largely addresses the spiritual/existential dimension, although when performed while walking it also can have a positive effect on the body's internal workings (e.g., the cardiovascular, gastrointestinal, and muscular systems). Walking, however, can be seen as addressing all four dimensions at once: it is clearly a physical activity with a variety of physical benefits; it affords both stimulation and quietude, especially when undertaken in natural surroundings, thus addressing the mental dimension by allowing the stressed portions of the brain to relax and reload; it can be practiced along with a partner, family member or friend, thus addressing the social dimension; and finally, it can afford one the opportunity to come in contact with one's chosen deity, which addresses the spiritual/existential dimension as well.

In this regard, the Austrian logotherapist Victor Frankl reminds us that individuals must be understood holistically as physical, psychological, social, and spiritual beings (McFadden et al., 2009). Following Frankl's lead, this article has attempted to contribute to the nursing profession by highlighting the importance of self-care management in terms of mindful body scanning, prayer, and meditative walking. As the world continues to grapple with the severity of the COVID-19 pandemic, it is our hope that the self-care suggestions contained in this article will be welcomed by nurses and their patients alike. It is our firm conviction that by implementing these self-care measures to whatever degree possible, nurses will be able to boost their physical energy, mental alertness, social cohesion, and sense of spiritual meaning, thus bringing greater empathy, compassion, and inner strength to their work.

## Notes

1. According to Wong and Wong (2012, p. 593), existential resilience are related to the quest of meaning and purpose in life. Existential resilience becomes spiritual resilience when the person resorts to religious or spiritual perspective to answer life's big questions.
